# Laser hemorrhoidoplasty versus hemorrhoidectomy in the treatment of surgically indicated hemorrhoids in inflammatory bowel patients: a randomized comparative clinical study

**DOI:** 10.1007/s00464-024-11351-3

**Published:** 2024-11-07

**Authors:** Reham Zakaria, Mohamed Mahmoud Amin, Heba Alhussein Abo-Alella, Yasmine Hany Hegab

**Affiliations:** 1 Department of General Surgery, College of Medicine, Zagazig University, Zagazig, Sharqia Egypt; 2 Department of Surgery, Zagazig University Hospitals, Zagazig, 44519 Sharqia Egypt

**Keywords:** Hemorrhoids, Inflammatory bowel, Crohn’s disease, Ulcerative colitis, Laser, Pain score

## Abstract

**Background:**

This study aims to assess and compare the outcomes of traditional surgery and laser surgery for anal hemorrhoids in patients with inflammatory bowel disease.

**Methods:**

This is a single-center prospective randomized comparative clinical trial performed at Zagazig University Hospitals from September 2023 to September 2024. The study comprised 48 patients who were admitted during this period and had inflammatory bowel disease along with hemorrhoids. The patients were randomly allocated into two groups: Group I consisted of 24 patients who underwent laser hemorrhoidoplasty, selected based on odd numbers, while Group II consisted of 24 patients who underwent traditional surgery, selected based on even numbers.

**Results:**

Group I, which underwent laser treatment for hemorrhoids in inflammatory bowel patients, demonstrated significantly better outcomes than traditional surgery (*p* < 0.001) regarding operation time, pain score and duration, postoperative anal discharge, return to work time, and patient satisfaction. Moreover, laser treatment was found to be more effective than traditional surgery for treating hemorrhoids in inflammatory bowel patients in terms of postoperative bleeding and anal stenosis. Additionally, there was a higher frequency of residual hemorrhoids in laser group.

**Conclusion:**

Laser treatment is mostly superior to traditional surgery for hemorrhoids in inflammatory bowel patients.

**Trial registration:**

Clinical Trial.gov (NCT06216223), registered on December 27, 2023, last updated in May 12, 2024.

**Protocol registration ID:**

#101080-5-9-2023

Perianal disease is a common complication associated with inflammatory bowel conditions, such as Crohn’s, ulcerative, or nonspecific colitis. The manifestations of this condition can vary greatly, encompassing both troublesome and annoying conditions such as fistulas and abscesses, as well as less severe disorders like hemorrhoids, skin tags, and fissures [1].

Due Anal sepsis and fistulae’s significant disease burden and ability to alter the disease’s progression have been the primary focus of attention in the literature. Despite their potential to impede a patient’s daily activities, hemorrhoids and anal fissures in individuals with inflammatory bowel disorders have been overlooked [2].

Given the potential for anal disease recurrence, surgeons initially opted for conservative medical treatment as the first choice in managing the course of inflammatory bowel disorders or related conditions. Furthermore, the results of surgical intervention were unsatisfactory, with a high likelihood of symptoms recurring or persisting. As a result, patients were required to continue taking immunosuppressive and corticosteroid medications after the surgery to prevent or slow down the progression of the inflammatory disease, which could potentially lead to delayed or no healing.

Currently, laser hemorrhoidoplasty (LHP) has garnered significant interest [3]. However, to our knowledge, there have been no published studies investigating the effectiveness of laser hemorrhoidoplasty compared to traditional surgery for patients with inflammatory bowel disease and severe anal disease.

Due to the prevalence of anal disease in individuals with inflammatory bowel disease and the challenges associated with treating anal problems in these patients, we aimed to examine a minimally invasive technique for treatment of the disease in patients requiring surgical intervention. Our objective was to compare the efficacy and complications of Laser ablation versus traditional surgery.

We hypothesize that Laser ablation is superior to surgery for hemorrhoids in IBD patients.

This study aims to improve the outcome of surgery in inflammatory bowel conditions.

## Materials and methods

Study design and population: This is a prospective comparative randomized parallel clinical trial, block randomization was done with an allocation ratio of 1:1 that involved 48 patients with inflammatory bowel conditions, including Crohn’s disease, ulcerative colitis, or nonspecific colitis, who had hemorrhoids. It included patients admitted during the period from September 2023 to September 2024.

Randomization and blinding: Participants were randomized into two groups using simple randomization. Every patient was assigned a number, and all numbers were placed into a container. Randomization was done by an assistant blind to the study procedures and group allocation. Group I (the odd numbers group) was treated with laser ablation, and Group II (the even numbers group) was treated with traditional surgery. Participants were blind to the selected procedures. Data were collected by a physician (health care provider) after surgery and in outpatients who was unaware of the procedures and the trial.

Sample size: Assuming that the mean score of the postoperative pain threshold was 5.3 ± 3.4 vs. 7.9 ± 5.9 in laser versus traditional surgery in anal fissures and hemorrhoids, at 80% power and 95% C, the required sample size for hemorroids was determined to be 48 cases (24 cases in each group)—open Epi.

The study was adherent to CONSORT 2010 guidelines with protocol registration ID: #101,080-5-9-2023 and registered at Clinical Trial.gov (NCT06216223).

Inclusion and exclusion criteria: The study included all patients of both sexes aged between 18 and 60 years with inflammatory bowel diseases (nonspecific colitis, Crohn’s disease, ulcerative colitis), controlled by medical treatment (corticosteroids and immunosuppressant drugs) or in the remission state along with hemorrhoids. All grades of hemorrhoids were included. Patients who refused to provide informed consent, proven malignancy in IBD, under 18 years old or more than 60 years old, had contraindications to general or spinal anesthesia, those with combined anal fissures and hemorrhoids, or those associated with fistula or abscess, as well as patients eligible for conservative treatment, were excluded from the study.

Indications for surgery included failure and non-compliance of medical treatment, severe pain and bleeding, and previous attacks of thrombosed hemorrhoids.

Perioperative and operative steps:

An outpatient with hemorrhoids underwent a pre-operative examination to assess the grade and number of hemorrhoids. Patients fasted for 6 h and were admitted for day-case surgery without bowel preparation. Patients received spinal anesthesia and were placed in the lithotomy position. Group I underwent laser hemorrhoidoplasty. Group II underwent ligation and excision for hemorrhoids (hemorrhoidectomy). All cases operated by the same surgeon.

Laser hemorrhoidoplasty was performed in pulsed mode using a curved laser fiber probe. Laser fiber was delivered directly to the hemorrhoidal node from outside at the mucocutaneous junction and then passed into the sub-mucosal plane until reaching the dentate line. The hemorrhoidal plexus was coagulated in a sequential manner using pulsed mode. Ice packs were applied to beamed areas, which had a cooling effect, resulting in reduced pain and edema. The shrinkage of hemorrhoids was noticed immediately [4]. Each hemorrhoid required approximately 250–300 J in pulsed mode. Anal packing was unnecessary.

The traditional surgery group underwent open hemorrhoidectomy (Milligan–Morgan) procedure which was performed by grasping the hemorrhoid externally using Allice forceps and removing it from the internal anal sphincter using monopolar diathermy until reaching the pedicle. The pedicle was then ligated with Vicryl 3\0. Anal packing was performed [5].

Intraoperatively, patients received a dose of corticosteroids and prophylactic antibiotics (ciprofloxacin and metronidazole) and continued to take them along with immunosuppressive therapy postoperatively.

Follow-up:

After discharge, patients were followed up as outpatients twice a week (for a month) and once monthly for six months or more frequently if they developed complications between their visits. Pre-, intra-, and postoperative data were recorded.

Outcome Measures: Age, sex, type of inflammatory bowel confirmed by colonoscopy and biopsy, and number and degree of hemorrhoids for the participants were examined and recorded two days before surgery. The operation time (in minutes) of the two groups, from skin incision to wound dressing, was recorded. Intraoperative bleeding was measured in mL using the gravimetric method. The procedure involved weighing surgical gauze and dressings soaked with blood and subtracting dry weight. The same method was used postoperatively by collecting pads or dressings worn by the patients and recording their number and weight every day for two weeks. Also, patients were in direct contact with the physician daily for visual estimation of the amount of blood loss. The blood loss was calculated with a conversion of 1 g = 1 ml [6]. All patients were categorized as class I (having ≤ 750 ml of blood loss) according to The American College of Surgeons Advanced Trauma Life Support (ATLS) hemorrhagic shock classification [7].

The pain score was assessed using a visual analog scale with a range of 0 to 10. A score of 0 means painless, whereas a maximum score of 10 indicates severe pain. The pain was assessed in the 1st, 2nd, 3rd, 7th, and 14th days, and the mean score was calculated. The duration of analgesia consumption in days was recorded for two weeks postoperatively. The presence of postoperative anal discharge and its duration in days were evaluated over a two-week period following the surgery.

Furthermore, time for complete healing in days was recorded from the first day to three months postoperatively by confirmed by digital rectal examination and proctoscopy, time to return to work was assessed from one day to two months after surgery, and patient satisfaction scores among patients were studied for three months postoperatively using the customer satisfaction score, where a minimum score of 1 means very dissatisfied and a maximum score of 10 means very satisfied.

The study examined the occurrence of recurrence between 3 and 6 months after the operation, while the occurrence of fistulas in was examined between 2 weeks and 2 months following the procedure. Stenosis was detected from wound healing to 6 months later, and residual hemorrhoids were evaluated from 2 to 6 months postoperatively.

Statistical design: The collected results were tabulated, summarized, and statistically analyzed using SPSS version 17. Standard deviation (SD), the Independent *t* test, and the Chi-square test (*χ*^2^) were used to compare the two groups.

## Results

Demographic, clinical, operative, and postoperative data for hemorrhoid cases are shown in Table 1.

**Table 1:** Demographic, clinical, operative, and postoperative data for hemorrhoid cases

Variable	Group A (Laser) (*n* = 24)		Group B (Surgery) (*n* = 24)		Test	*p*
	No	%	No	%		
*Age: (years)*						
Mean ± Sd	38.67 ± 6.91		35.21 ± 7.02		t	
Range	26–50		21–48		1.72	0.09 NS
*Sex*						
Female	13	54.2	14	58.3	*χ*^2^	
Male	11	45.8	10	41.7	0.09	0.77 NS
*Disease*						
Colitis	11	45.8	11	45.8	*χ*^2^	
Crohn’s	6	25	6	25	0	
Ulcerative	7	29.2	7	29.2		1 NS
*Degree*						
2nd	6	25	5	20.8	*χ*^2^	
3rd	11	45.8	12	50	0.13	
4th	7	29.2	7	29.2		0.94 NS
*Number*						
1	3	12.5	1	4.2	*χ*^2^	
2	8	33.3	9	37.5	2.21	
3	12	50	14	58.3		
4	1	4.2	0	0		0.53 NS
*Operation time: (min)*						
Mean ± Sd	12.67 ± 2.28		27.5 ± 7.51		*t*	
Range	10–18		15–40		9.26	< 0.001**
*Bleeding intra operation*						
No	20	83.3	14	58.3	*χ*^2^	
Yes	4	16.7	10	41.7	3.63	0.06 NS
*Reactionary Hg*						
No	23	95.8	18	75	*χ*^**2**^	
Yes	1	4.2	6	25	4.18	0.04*
*2ry Hg*						
No	24	100	13	54.2	*χ*^**2**^	
Yes	0	0	11	45.8	14.27	< 0.001**
*Pain score*						
Mean ± Sd	4 ± 0.98		7.75 ± 1.19		*t*	
Range	2–6		6–10		11.93	< 0.001**
*Analgesia duration (day)*						
Mean ± Sd	2.13 ± 0.74		4.58 ± 0.83		*t*	
Range	1–3		3–6		10.83	< 0.001**
*Anal discharge duration (day)*						
Mean ± Sd	5 ± 2.69		6.63 ± 3.76		MW	
Median	4		7		1.98	
Range	2–11		1–13			0.04*
*Healing duration: (day)*						
Mean ± Sd	13.79 ± 2.28		38.63 ± 11.36		*t*	
Range	10–20		20–60		10.50	< 0.001**
*Return to work: (day)*						
Mean ± Sd	9.83 ± 4.03		27.83 ± 5.77		*t*	
Range	4–17		21–38		12.53	< 0.001**
*Fistula*						
No	20	83.3	23	95.8	*χ*^2^	
Yes	4	16.7	1	4.2	2.01	0.16 NS
*Recurrence*						
No	18	75	18	75	*χ*^2^	
Yes	6	25	6	25	0	1 NS
*Residual hemorrhoids*						
No	20	83.3	24	100	*χ*^**2**^	
Yes	4	16.7	0	0	4.36	0.04*
*Patient satisfaction score*						
Mean ± Sd	7.54 ± 0.93		5.29 ± 1.08		*t*	
Range	6–9		3–7		7.72	< 0.001**
*Stenosis*						
No	24	100	11	45.8	*χ*^**2**^	
Yes	0	0	13	54.2	17.83	< 0.001**

*SD* Standard deviation, *t* Independent t test, *χ*^2^ Chi-square test, *MW* Mann–Whitney test, *Hg* Hemorrhage

*NS* Non-significant (*p* > 0.05), *Significant (*p* < 0.05), **Highly significant (*p* < 0.001)

Overall, there were no differences between both groups regarding age, sex, inflammatory disease presentation, number, degree of hemorrhoids, intraoperative bleeding, postoperative fistula, or recurrence. The laser group exhibited a substantial difference in operation time, secondary hemorrhage, pain score, duration of analgesia, healing duration, return to work, patient satisfaction, and postoperative anal stenosis, with a *p* value < 0.001. Reactionary hemorrhage and postoperative anal discharge were significantly lower in the laser group, while postoperative residual hemorrhoids were significantly lower in the surgery group.

Operative and postoperative data for hemorrhoid cases in nonspecific colitis are displayed in Table 2. Operation time, pain score, analgesia duration, healing time, return to work, and patient satisfaction score were significantly better in the laser group, with a *p* value < 0.001. Secondary hemorrhage and postoperative stenosis were lower in the laser group, with a *p* value < 0.05. Conversely, there was no significant difference in reactionary hemorrhage, postoperative anal discharge, postoperative fistula, recurrence, or residual hemorrhoids between the two groups.

**Table 2:** Operative and postoperative data for hemorrhoid cases in nonspecific colitis

Variable	Group A (Laser) (*n* = 11)		Group B (Surgery) (*n* = 11)		Test	*p*
	No	%	No	%		
*Operation time: (min)*						
Mean ± Sd	12.18 ± 1.89		21.82 ± 6.81		*t*	
Range	10–15		15–30		4.52	< 0.001**
*Bleeding intra operation*						
No	9	81.8	7	63.6	*χ*^2^	
Yes	2	18.2	4	36.4	0.92	0.34 NS
*Reaction Hg*						
No	11	100	8	72.7	*χ*^2^	
Yes	0	0	3	27.3	3.47	0.06 NS
*2ry Hg*						
No	11	100	7	63.6	*χ*^**2**^	
Yes	0	0	4	36.4	4.89	0.03*
*Pain score*						
Mean ± Sd	4.18 ± 1.17		7.45 ± 1.29		*t*	
Range	2–6		6–10		6.23	< 0.001**
*Analgesia duration (day)*						
Mean ± Sd	1.91 ± 0.70		4.55 ± 0.69		*t*	
Range	1–3		4–6		8.91	< 0.001**
*Anal discharge duration (day)*						
Mean ± Sd	3.91 ± 1.76		3 ± 1.48		t	
Range	2–7		1–5		1.31	0.21 NS
*Healing time: (day)*						
Mean ± Sd	12.36 ± 1.75		31.36 ± 7.69		*t*	
Range	10–15		20–45		8	< 0.001**
*Return to work: (day)*						
Mean ± Sd	6 ± 1.10		25.18 ± 4.62		*t*	
Range	4–7		21–30		13.39	< 0.001**
*Fistula*						
No	10	90.9	11	100	*χ*^2^	
Yes	1	9.1	0	0	1.05	0.31 NS
*Recurrence*						
No	9	81.8	10	90.9	*χ*^2^	
Yes	2	18.2	1	9.1	0.39	0.53 NS
*Residual hemorrhoids*						
No	10	90.9	11	100	*χ*^2^	
Yes	1	9.1	0	0	1.05	0.31 NS
*Patient satisfaction score*						
Mean ± Sd	7.73 ± 1.01		5.73 ± 0.65		*t*	
Range	6–9		5–7		5.54	< 0.001**
*Stenosis*						
No	11	100	5	45.5	*χ*^**2**^	
Yes	0	0	6	54.5	8.25	0.004*

*SD* Standard deviation, *t* Independent t test, *χ*^2^ Chi-square test, *Hg* Hemorrhage

*NS* Non-significant (*p* > 0.05), *Significant (*p* < 0.05), **Highly significant (*p* < 0.001)

Table 3 illustrates the intra- and postoperative outcomes for hemorrhoid cases between the two groups of patients with Crohn’s disease. The laser group demonstrated significantly reduced operation time, lower pain scores, faster healing time, earlier return to work, and increased patient satisfaction (*p* value < 0.001). Furthermore, the laser group exhibited a lower secondary hemorrhage, lower postoperative stenosis, and shorter analgesia duration (*p* value < 0.05). However, there was no difference in intraoperative bleeding, anal discharge, postoperative fistula, recurrence, or residual hemorrhoids between the two groups.

**Table 3:** Intra- and postoperative outcomes for hemorrhoids’ cases in Crohn’s disease

Variable	Group A (Laser) (*n* = 6)		Group B (Surgery) (*n* = 6)		Test	*p*
	No	%	No	%		
*Operation time: (min)*						
Mean ± Sd	13 ± 2.97		32.67 ± 3.78		*t*	
Range	10–18		28–37		10.03	< 0.001**
*Bleeding intra operation*						
No	5	83.3	3	50	*χ*^2^	
Yes	1	16.7	3	50	1.5	0.22 NS
*Reaction Hg*						
No	6	100	3	50	*χ*^**2**^	
Yes	0	0	3	50	4	0.04*
*2ry Hg*						
No	6	100	3	50	*χ*^**2**^	
Yes	0	0	3	50	4	0.04*
*Pain score*						
Mean ± Sd	4.17 ± 0.98		8.33 ± 1.03		*t*	
Range	3–5		7–10		7.16	< 0.001**
*Analgesia duration (day)*						
Mean ± Sd	2.33 ± 0.82		4.83 ± 1.17		*t*	
Range	1–3		3–6		4.29	0.002*
*Anal discharge duration (day)*						
Mean ± Sd	8.5 ± 2.42		10.33 ± 1.03		t	
Range	5–11		9–12		1.70	0.12 NS
*Healing time: (day)*						
Mean ± Sd	15.17 ± 2.71		51 ± 7.32		*t*	
Range	12–20		41–60		11.24	< 0.001**
*Return to work: (day)*						
Mean ± Sd	11.33 ± 1.51		34.17 ± 3.37		*t*	
Range	10–14		30–38		15.15	< 0.001**
*Fistula*						
No	4	66.7	5	83.3	*χ*^2^	
Yes	2	33.3	1	16.7	0.44	0.51 NS
*Recurrence*						
No	4	66.7	3	50	*χ*^2^	
Yes	2	33.3	3	50	0.34	0.56 NS
*Residual hemorroids*						
No	5	83.3	6	100	*χ*^2^	
Yes	1	16.7	0	0	1.09	0.30 NS
*Patient satisfaction score*						
Mean ± Sd	6.83 ± 0.75		3.83 ± 0.75		*t*	
Range	6–8		3–5		6.90	< 0.001**
*Stenosis*						
No	6	100	2	33.3	*χ*^**2**^	
Yes	0	0	4	66.7	6	0.01*

*SD* Standard deviation, *t* Independent t test, *χ*^2^ Chi-square test, *MW* Mann–Whitney test, *Hg* Hemorrhage

*NS* Non-significant (*p* > 0.05), *Significant (*p* < 0.05), **Highly significant (*p* < 0.001)

Data for hemorrhoid cases among ulcerative colitis patients are shown in Table 4. There was no difference in intraoperative bleeding, reactionary hemorrhage, postoperative fistula, recurrence, or residual hemorrhoids. Secondary hemorrhage, anal discharge, and postoperative stenosis were significantly lower in the laser group, with a *p* value < 0.05. Additionally, operation time, analgesia duration, healing time, and pain score were lower. Within the laser group, return to work was earlier, and the score for patient satisfaction was higher.

**Table 4:** Intra- and postoperative outcomes for hemorrhoids cases in ulcerative colitis

Variable	Group A (Laser) (*n* = 7)		Group B (Surgery) (*n* = 7)		Test	*p*
	No	%	No	%		
*Operation time: (min)*						
Mean ± Sd	13.14 ± 2.41		32 ± 4.20		*t*	
Range	10–16		28–40		10.30	< 0.001**
*Bleeding intra operation*						
No	6	85.7	4	57.1	*χ*^2^	
Yes	1	14.3	3	42.9	1.4	0.23 NS
*Reaction Hg*						
No	6	85.7	7	100	*χ*^2^	
Yes	1	14.3	0	0	1.08	0.30 NS
*2ry Hg*						
No	7	100	3	42.9	*χ*^**2**^	
Yes	0	0	4	57.1	5.6	0.02*
*Pain score*						
Mean ± Sd	3.57 ± 0.54		7.71 ± 1.11		*t*	
Range	3–4		6–9		8.88	< 0.001**
*Analgesia duration (day)*						
Mean ± Sd	2.29 ± 0.76		4.43 ± 0.79		*t*	
Range	1–3		3–5		5.20	< 0.001**
*Anal discharge duration (day)*						
Mean ± Sd	3.71 ± 1.11		9.14 ± 2.12		*t*	
Range	2–5		7–13		6.01	0.04*
*Healing time: (day)*						
Mean ± Sd	14.86 ± 1.35		39.43 ± 10.03		*t*	
Range	13–17		30–60		6.42	< 0.001**
*Return to work: (day)*						
Mean ± Sd	14.57 ± 1.72		26.57 ± 5.26		*t*	
Range	12–1		21–35		5.74	< 0.001**
*Fistula*						
No	6	85.7	7	100	*χ*^2^	
Yes	1	14.3	0	0	1.08	0.30 NS
*Recurrence*						
No	5	71.4	5	71.4	*χ*^2^	
Yes	2	28.6	2	28.6	0	1 NS
*Residual hemorrhoids*						
No	5	71.4	7	100	*χ*^2^	
Yes	2	28.6	0	0	2.33	0.13 NS
*Patient satisfaction score*						
Mean ± Sd	7.86 ± 0.69		5.86 ± 0.69		*t*	
Range	5–7		7–9		5.42	< 0.001**
*Stenosis*						
No	7	100	4	57.1	*χ*^**2**^	
Yes	0	0	3	42.9	3.82	0.05*

*SD* Standard deviation, *t* Independent t test, *χ*^2^ Chi-square test, *MW* Mann–Whitney test, *Hg* Hemorrhage

*NS* Non-significant (*p* > 0.05), *Significant (*p* < 0.05), **Highly significant (*p* < 0.001)

## Discussion

Anal fissures and hemorrhoids are common issues in patients with inflammatory bowel. Several studies have examined the efficacy of laser hemorrhoidoplasty. However, no studies or research have discussed laser ablation for patients with inflammatory bowel. The management of anal problems in these patients is challenging, as surgical treatment may have complications and exacerbate the disease’s burden. However, conservative management of not-indicated cases exacerbates the condition.

Diode lasers, carbon dioxide, argon, and Nd:YAG are the most prevalent types of laser energy utilized in the medical field. The laser beam results in tissue shrinkage and degeneration at varying degrees, depending on the laser strength and the duration of the laser light application. Recently, diode laser treatment has emerged as a new minimally invasive and painless method that is considered an alternative to surgery. It is associated with an early recovery, reduced bleeding, and less postoperative pain [8]. Laser techniques used for the treatment of hemorrhoidal disease were laser open hemorrhoidectomy, laser hemorrhoidoplasty, and Doppler-guided hemorrhoidal laser de-arterialization [9].

A total of 48 inflammatory bowel patients with hemorrhoids were admitted. 24 in each group (11 nonspecific colitis, 6 Crohn’s disease, and 7 ulcerative colitis). All patients were on corticosteroids and immunosuppressive therapy. There were no statistically significant differences between the two groups in age, sex, inflammatory disease distribution, degree, or number of hemorrhoids.

Operative duration was significantly shorter in the laser group than in the surgery group. This result was similar to Wee et al.’s study on normal individuals in 2023 [10], as the laser delivered high energy in a shorter time compared to surgery besides, laser bypassed the time needed for pedicle ligation.

Torrinha et al. [11] illustrated that laser surgery is more hemostatic. In our study, we found no statistical difference between the two groups in intraoperative bleeding due to fibrosis. Nevertheless, reactionary and secondary hemorrhage was significantly lower in the laser group than in the surgery group, as lasers cause coagulation of the venous plexus.

For nonspecific colitis and ulcerative colitis cases, there was no difference between laser and surgery in reactionary hemorrhage. In contrast, secondary hemorrhage was less common in the laser group. Reactionary and secondary hemorrhage were less in laser than in the surgery group for Crohn’s disease patients. The presence of anal disease in Crohn’s disease can be linked to increased inflammation, as it is an inherent aspect of the disease pathology.

Maloku and his colleagues [12] found that the pain scores were significantly lower in the laser hemorrhoidoplasty group compared with the open hemorrhoidectomy procedure group in healthy patients, which is consistent with our study. This can be explained by the reduction in inflammatory mediators, such as TNF-*α* and IFN-*γ* [13], intact mucosa, venous coagulation in laser, and wide raw areas in surgery.

We assessed postoperative pain using a visual analog scale. This showed that the laser group had significantly lower pain scores. This agrees with Shabahang et al.’s study [14], and the duration of analgesia was shorter in the laser group due to reducing inflammation.

Anal discharge is a common and annoying postoperative sequel after hemorrhoidectomy. Anal discharge duration was statistically shorter in the laser group, especially in ulcerative colitis patients, due to intact mucosa. Patients with laser hemorrhoidoplasty returned earlier to work than the surgery group. Complete healing was faster in the laser group. There was no statistical difference in fistula occurrence between the two groups. There was no statistical difference regarding recurrence after six months of follow-up, but the surgery group was better at leaving no residual hemorrhoids, as some laser patients needed a second session of hemorrhoidoplasty or mucopexy especially in grade 4. Overall, patient satisfaction was assessed after three months by the patient satisfaction score. The results revealed that laser group patients demonstrated higher satisfaction than surgery group patients.

Postoperative anal stenosis assessed six months following surgery was higher among the surgery group. Cases with nonspecific colitis, Crohn’s disease, or ulcerative colitis had no stenosis in the laser group. However, in the surgical group, 60% of cases with nonspecific colitis, 66.6% of cases with Crohn’s disease, and 43% of cases with ulcerative colitis had stenosis. Excessive removal of the mucosal lining is the primary cause of postoperative anal stenosis. Circumferential removal of mucosa, fibrosis, and stricture which are a part of the disease progression, delayed healing or non-healing which are very common due to systemic and local corticosteroids and immusupressive drugs, sphincterotomy which improve the healing and fibrosis is contraindicated in IBD are other causes. Due to the preservation of the anal mucosa after laser ablation, there was no occurrence of stenosis. Since fibrosis and necrosis in the anal mucosa are more common in Crohn’s disease, inflammation and stenosis was higher in this group, followed by nonspecific colitis then ulcerative colitis.

## Conclusion

Laser hemorrhoidoplasty demonstrated better outcomes compared to traditional surgery in treating hemorrhoids in inflammatory bowel conditions, such as nonspecific colitis, Crohn’s disease, and ulcerative colitis. The laser group had a shorter operative time, lesser bleeding, pain and analgesia duration, anal discharge, and postoperative anal stenosis. It also had faster healing and returned to work with a higher patient satisfaction score, but it left more residual hemorrhoids.

## Data Availability

No datasets were generated or analyzed during the current study.

## Declarations

### Trial limitations and recommendations

We suggest that future studies extend the minimum follow-up period to evaluate long-term results, including the likelihood of long-term recurrence and to evaluate a larger number of cases due to the scarcity of IBD cases.
